# Promoting emotional & behavioral health for pediatric patients with Fontan circulation: integrating psychology into a dedicated multidisciplinary clinic

**DOI:** 10.3389/fcvm.2025.1623352

**Published:** 2025-07-11

**Authors:** Nicholas P. Seivert, Kathryn M. Dodds, Abigail Demianczyk, David J. Goldberg, Jack Rychik

**Affiliations:** ^1^ Department of Child & Adolescent Psychiatry & Behavioral Sciences, Children’s Hospital of Philadelphia, PA, United States; ^2^ Perelman School of Medicine, University of Pennsylvania, Philadelphia, PA, United States; ^3^ School of Nursing, University of Pennsylvania, Philadelphia, PA, United States; ^4^ Cardiac Center, Children’s Hospital of Philadelphia, Philadelphia, PA, United States; ^5^ Children’s Behavioral Health and Neurosciences, Cleveland Clinic, Cleveland, OH, United States

**Keywords:** single ventricle, mental health, congenital heart disease, integrated care, program development

## Abstract

**Introduction:**

Individuals with single ventricle congenital heart disease and Fontan circulation are at high risk for mental health problems. There is a lack of information about potential care models to address these common challenges. This study describes the innovative integration of psychology consultations into a multidisciplinary clinic for pediatric patients with Fontan circulation. Findings from emotional/behavioral and quality-of-life measures are reported and relationships between these variables are explored.

**Method:**

The Fontan Rehabilitation, Wellness and Resilience Development (FORWARD) Program at Children's Hospital of Philadelphia is a multidisciplinary clinic for individuals with Fontan circulation. A psychologist provides behavioral health consultations to all clinic patients. The psychologist engages in real-time care coordination and treatment planning with the multidisciplinary team. Families are administered standardized screening questionnaires assessing child emotional/behavioral functioning (Behavior Assessment System for Children-3) and quality-of-life (Pediatric Cardiac Quality of Life Inventory). The psychologist provides brief behavioral intervention and recommendations for follow up care. Patient data were gathered by chart review. Correlations explored the relations between selected screening measures.

**Results:**

158 patients (mean age = 12.5 years) were seen between January 2019 – June 2022. 92% completed a psychology consultation. Most had hypoplastic left heart syndrome (54%), were male, White, and had commercial insurance. A majority completed at least one of the screening measures. Elevated symptoms were found for 23% of the sample for depression/anxiety, 37% for inattention, and 22% reported school problems. Findings from the quality-of-life measure showed mild to moderate impairment for the sample. Greater inattention, depression/anxiety, and school problems correlated with lower quality-of-life scores.

**Discussion:**

This study demonstrates the feasibility of innovatively implementing regular psychology consultations into a multidisciplinary clinic for children and adolescents with Fontan circulation. Greater emotional/behavioral problems were associated with poorer quality-of-life. Fully integrated care models that include including behavioral screening measures are optimal to address emotional/behavioral challenges in this population. Less resource intensive models could be implemented as pilot programs to establish feasibility and utility. Research is needed to evaluate efficacy of such programs and examine potential links between medical and psychological variables.

## 1 Introduction

Children with single ventricle (SV) congenital heart disease (CHD) require multiple surgical interventions during the first few years of life, culminating with the Fontan procedure. Most patients with Fontan circulation (FC) live well into adulthood, but remain at high risk for physical, neurodevelopmental, and psychological comorbidities, requiring lifelong monitoring. The American Heart Association developed guidelines for evaluation and management of children with FC, including recommendations regarding neurodevelopmental and emotional/behavioral healthcare to address the common developmental and psychological concerns seen in this population (1). For example, one study found a lifetime prevalence rate of 65% for mental health disorders in adolescents with SV CHD compared to 22% in the control sample (2). Individuals with FC also demonstrate impairments in health-related quality of life (HRQOL), with a recent meta-analysis noting the greatest impact in the areas of physical and occupational (school/work) functioning (3).

Although some pediatric heart centers have described generally how psychosocial providers (e.g., social workers, psychologists) are included as part of their care teams to address these needs (4–6), there is a lack of detailed description of the process for how to incorporate these clinicians into routine care specifically for SV CHD. Another key gap in the literature is determining how systematic screening of school-age and adolescent patients could be implemented in the context of specialized multidisciplinary programs caring for these patients (4).

This report provides descriptive information regarding how routine psychological screening and consultation with a pediatric psychologist can be innovatively integrated into a multidisciplinary clinic for children and adolescents with FC. The study presents patient demographic, mental health, and quality of life data collected as part of the psychology consultation in this specialty clinic. The papers explores associations between mental health and quality of life measures completed by patients and caregivers. Practice implications and recommendations for psychological screening in this population, including suggestions for implementation into routine care, are discussed.

## 2 Methods

### 2.1 Development of the multidisciplinary clinic

In 2011, the Cardiac Center at Children's Hospital of Philadelphia established what was then called the Single Ventricle Survivorship Program, a clinic to provide specialized evaluation and monitoring for children and adolescents with FC. The clinic initially included a team of medical providers tailored to the specific medical needs of this population, including cardiology, gastroenterology/hepatology, immunology, and endocrinology. During this initial phase, program leadership recognized the significant psychological burden and quality of life impacts for patients and families related to managing their SV CHD. This became apparent not only based on clinical presentations of patients but also specific feedback from families and emerging research on the unique needs of this population. In 2017 the clinic evolved becoming the Fontan Rehabilitation, Wellness and Resilience Development (FORWARD) Program, expanding the multidisciplinary team to include a dietician, exercise physiologist, social worker, and psychologist. A general overview of the program, its development, and summary of the approach to evaluation for each specialty is described in Rychik et al. (5). FORWARD program services are consultative and generally do not replace the role of the primary cardiologist but are rather additive to established care. To be part of the program, patients are either self-referred or referred by their primary cardiologist. Both patients from the program's home institution, Children's Hospital of Philadelphia, as well as those that are followed by outside cardiologists from across the country are eligible to participate.

Given the significant risk for a variety of cognitive, social, emotional, and behavioral challenges in patients with FC, FORWARD Program leadership recruited a dedicated pediatric psychologist to provide evaluations for each clinic patient. There are a variety of models for integration of psychologists and other mental health professionals into pediatric medical settings, with emerging evidence showing improved patient outcomes as a result (7). Program leadership determined that a fully integrated care model would best meet the needs of patients. This involves the psychologist meeting with every clinic patient and their family for a behavioral health consultation and administration of psychological screening measures, further described below. Psychology participates in clinic rounds with the other multidisciplinary team members for coordination of care and integrated treatment planning. In addition to clinical evaluations and care coordination, the program provides protected time for the psychologist to engage in program development and research, a key factor that has allowed for the implementation of quality improvement initiatives and dissemination of findings from this novel program. In addition, psychology collaborates with social work who meets with each patient and family to provide assessment and support around basic needs (e.g., housing, food, insurance, finances, school) as well as connection with appropriate resources to address any identified concerns.

### 2.2 Approach to psychology consultation

Prior to the clinic visit, families are mailed informational materials by the program coordinator regarding the elements of the clinic, which includes psychology consultation as a component of the multidisciplinary assessment. This information is also posted on the program website. Nearly all families in the program are open to the psychology consultation. Very rarely are patients not seen by psychology, and if not, it is typically because of insurance coverage barriers. Although uncommon, a very small number of families decline psychology consultation, even after discussion with the psychologist, program coordinator, or clinic manager about any issues they may have concerning the need to meet with a psychologist.

On a separate day prior to the clinic date, patients come to the hospital to complete testing in preparation for meeting with providers including blood labs draw, electrocardiogram, echocardiogram, exercise testing, and bone density scan, as per age-based care recommendations made by the AHA (1). When routine psychology consultations were initially implemented in the program, patients were typically seen by psychology during the course of their testing day, and if not, psychology would see them on their clinic day along with the other providers. Now, due to the increased use of telehealth since the COVID-19 pandemic, many patients are seen for their psychology visit virtually using the medical record's telehealth platform at a time scheduled prior to their clinic day.

The program psychologist meets with each FORWARD Clinic patient and family for clinical assessment and administration of psychological screening measures. The domains assessed during the clinical interview with patients and caregivers include mental health history, current psychological concerns, educational, social, developmental, and family history, understanding of medical recommendation including medication adherence, patient knowledge about their medical history, and coping with medical cares and procedures. Patients ages 12 years and older are given the opportunity to meet alone with the psychologist if they would like. Each child and parent are also asked to complete standardized psychological screening questionnaires. All children ages 8–21 years, the typical age range of patients seen in the clinic, and their caregivers are asked to complete the Behavioral Assessment Scale for Children, Third Edition (BASC-3) (8), a broad screening measure that assesses a variety of common mental health concerns and provides results in T-scores by comparing patient scores to the standardization sample based on age. Also administered is the Pediatric Cardiac Quality of Life Inventory (PCQLI) (9), a health-related quality of life measure specific to pediatric cardiac populations. Based on guidelines from the authors of the measures, patients ages 8–18 years and their caregivers are administered child self-report and parent report forms, respectively, of the PCQLI. Measures are sent to families electronically for completion prior to their appointment with the psychologist, taking approximately 20–30 min for each informant to complete. If the patient has significant cognitive or learning challenges, only caregiver report measures are administered.

At the end of the evaluation, the psychologist provides recommendations to patients and their families based on concerns identified through the clinical interview and results from psychometric screenings. Common recommendations include: (1) referrals for additional mental health services, such as psychotherapy, psychotropic medication evaluation with their pediatrician or referral to psychiatry, and neuropsychological testing, (2) suggestions for school-related accommodations and interventions, (3) strategies to improve medication adherence, and (4) developmentally sensitive strategies for increasing the patient's knowledge, coping, and general management of their health condition. The psychologist bills for consultations as a “Health and Behavior Assessment” (CPT Code: 96156) under the patient's primary cardiac diagnosis, which is typically covered by most public and commercial health insurance plans.

## 3 Results

The sample included all FORWARD Clinic patients seen between January 2019 and June 2022 (*n* = 158). The vast majority of clinic patients (92%) were seen by psychology for routine consultation during this time period. Most patients were male, identified their race as White, and had commercial insurance (see Table 1 for additional demographic information). The most common diagnosis was hypoplastic left heart syndrome (54%). The mean age for patients was 12.5 years (SD = 2.5). Participants were all clinically well outpatients coming for elective FORWARD evaluation. As for general health metrics, mean age-referenced height *z*-score was −0.8 (SD = 1.5), BMI was 20.0 (SD = 6.4), and resting oxygen saturation at the time of the clinic visit was 93.8% (SD = 3.0).

**Table 1:** Patient demographic information.

Variable		*N* (% of sample)
Sex	Male	95 (60.1)
	Female	63 (39.9)
Race	White	130 (82.3)
	Black/African American	4 (2.5)
	Asian	2 (1.3)
	Other/unknown	22 (13.9)
Ethnicity	Non-Hispanic/Latino	138 (87.4)
	Hispanic/Latino	15 (9.5)
	Unknown	5 (3.2)
Insurance status	Private/commercial	121 (76.6)
	Medicaid	27 (17.1)
	Other/unknown	10 (6.3)
Primary cardiac diagnosis	Hypoplastic left heart syndrome	85 (53.8)
	Double inlet left ventricle	14 (8.9)
	Other single ventricle CHD	59 (37.3)
Total *N*		158

Measures of psychological assessment were available for a portion of the patients seen by psychology. These measures were administered more consistently over time, eventually given to all families as a routine part of the consultation. Some families did not complete the measures or partially completed them despite being administered. Reasons as to why patients and/or parents did not complete the measures were not specifically assessed. For the general emotional/behavioral screening measure (BASC-3), 68% of patients (*n* = 107) had a parent report form completed and 51% (*n* = 80) had a child self-report completed. Data on the role of caregiver/parent completing the form (e.g., mother, father, grandmother, etc.,) and other caregiver background information were not collected during the study period. The BASC-3 assesses a wide variety of domains; scores from these measures (Table 2) were selected for reporting in this study based on the common concerns seen in patients with FC, that is, Internalizing (depression/anxiety symptoms), Attention Problems, and School Problems (child-report only). Of those with completed parent report measures, 23% showed elevated levels of depression/anxiety symptoms and 37% were elevated for attention problems. As for child self-report, 16% had elevated levels of depression/anxiety, 26% reported elevated attention problems, and 22% indicated elevated school problems. For the quality-of-life measure (PCQLI), parent report on child functioning was completed for 62% (*n* = 90) of patients and child self-report was completed for 52% (*n* = 75). Sample means for the PCQLI for parent and child are reported in Table 2. Results on all PCQLI scales were consistent across respondents and suggested mild to moderate impairment for the sample.

**Table 2:** Psychosocial measures.

Measure	Scale	Mean (SD)	*N* (%) Elevated scores
BASC-3 parent report	Internalizing	53.7 (69.1)	25 (23.4)
	Attention problems	54.8 (12.0)	40 (37.4)
BASC-3 child report	Internalizing	49.0 (10.4)	13 (16.3)
	Attention problems	51.8 (11.9)	21 (26.3)
	School problems	49.3 (12.8)	17 (21.5)
PCQLI parent report	Disease impact	32.3 (7.9)	–
	Psychosocial impact	35.0 (8.1)	–
	Total score	67.3 (14.6)	–
PCQLI child report	Disease impact	32.9 (9.1)	–
	Psychosocial impact	34.2 (8.8)	–
	Total score	67.2 (16.7)	–

Examinations of the relationship between BASC-3 and PCQLI data are presented in Table 3. For parent report of child functioning, PCQLI Total Score was negatively correlated with BASC-3 Internalizing and Attention Problems Scales, indicating those with lower quality of life scores had significantly greater depression/anxiety and inattention symptoms. Similarly, for child self-report, PCQLI Total Score was negatively associated with BASC-3 Internalizing, Attention, and School Problems Scales. Children who reported poorer quality of life indicated more problems with depression/anxiety, inattention, and school concerns.

**Table 3:** Correlations between PCQLI and BASC-3 scores.

PCQLI	BASC-3	*R*	95% CI		*P*
			Lower	Upper	
Parent total score	Internalizing	−.626	−.745	−.468	<.001
	Attention problems	−.465	−.623	−.270	<.001
Child total score	Internalizing	−.741	−.839	−.595	<.001
	Attention problems	−.443	−.631	−.207	<.001
	School problems	−.342	−.553	−.089	.009

## 4 Discussion

We demonstrate the feasibility of innovatively incorporating routine consultation with a pediatric psychologist into multidisciplinary teams caring for children and adolescents with FC, addressing a key gap in the literature regarding models of how to provide the needed psychological support to these individuals and families (4). There are a few key points to highlight for institutions considering implementing or expanding emotional and behavioral healthcare for this population. Collecting data through screening measures provides a standardized way to assess key domains of functioning relevant to the unique needs of this population. They are an important component of a thorough clinical consultation with a psychologist or other psychosocial provider. Our program chose to administer the BASC-3 because it assesses emotional/behavioral concerns in a wide variety of domains. Authors chose to report data from the scales most relevant to common concerns seen in FC in the current paper with the aim of streamlining how our findings are reported. The BASC-3 is a lengthy measure and test developers estimate it can take up to 30 min for respondents to complete. As an alternative, other programs may want to consider using briefer, more focused measures that are tailored to commonly occurring concerns, although this would result in losing the thoroughness of a more comprehensive measure. In addition, there are some models of care in which screening measures are administered universally to all patients and those reporting elevated levels of concerns are then referred to psychology. This method could be used to create a pilot behavioral health assessment, particularly for programs who have limited resources. Ideally, given the accumulating evidence on the high risk for cognitive, emotional, and behavioral concerns of this population, we recommend screening measures be administered universally in conjunction with routine psychology clinical consultation for all patients, as opposed to determining whether a consultation is needed.

Another important component is the use of a truly integrated model of care. The FORWARD Program psychologist participates in all clinic rounds, providing a summary of their assessment along with other clinic providers, and engages in live care coordination and treatment planning with the rest of the team. In other words, at these clinic rounds, the psychologist receives information concerning the medical aspects of the evaluation (e.g., need for additional cardiac procedures, liver fibrosis, endocrinological problems, immunological status), while each of the medical specialty providers also hears about the psychological assessment. This allows for incorporating psychological factors into the clinical conceptualization of each patient, providing for a comprehensive patient characterization signature, with the goal of improving the quality of care provided.

Due to clinical capacity and sometimes distance of a family's home from the clinic, the program psychologist is not able to regularly follow all patients that need emotional/behavioral health intervention services after the clinic consultation. The psychologist, often in collaboration with social work, provides local referrals for these services as needed and sometimes engages in care coordination for families outside of the actual clinic visit. For example, patients or caregivers may wish to follow up on the recommendations discussed during the consultation, such as through phone call or secure message in the medical record. Some families request additional guidance after the visit on seeking out the recommended supports, such as connecting with appropriate local mental health clinics or communication with the child's school regarding implementing academic accommodations or interventions. Patients requiring serial follow up care that could continue to be seen by the program psychologist live locally and are followed by a cardiologist at the program's home institution. Also, these patients typically have referral concerns related to their medical issues that would be best served by specialized care from a pediatric psychologist such as poor medication adherence, medical trauma, self-management of medical care when transitioning to adulthood, and significant anxiety about medical procedures (e.g., needle phobia, fears about upcoming catheterization, surgery, MRI, liver biopsy). These individuals are followed by the program psychologist typically for brief psychotherapy (approximately 4–10 sessions but can vary depending on need) using behavioral and cognitive-behavioral intervention strategies targeted at specific referral concerns related to their medical condition. Local patients with more general emotional/behavioral concerns, such as generalized anxiety, depression, and disruptive behavior problems, are most often referred to community mental health providers. Also, those individuals followed by the program psychologist for brief psychotherapy focused on medically-related issues that require additional or more ongoing support for general emotional/behavioral concerns may be referred for follow up in the community. The program's home institution has additional behavioral health resources to which patients can be referred as needed, such as neuropsychological testing, Autism-related care, and a psychiatry clinic focusing on individuals with comorbid medical and psychiatric issues.

We also would like to highlight the importance of flexibility in terms of scheduling and the methods used for completing the psychology evaluation. As a result of the COVID-19 pandemic, the program adopted an approach in which many families can engage in a telehealth consultation with the psychologist at a scheduled time prior to the on-site clinic visit. This allows for reduced burden for both families and the multidisciplinary team during the busy clinic day when patients are seen by other providers. Some patients are still seen on site during their testing day, as had previously been the clinic model. If scheduling conflicts or the need for a prior-authorization from insurance arises, patients are seen in person on the clinic day. In addition, patients with psychiatric complexity are often seen on clinic day to support optimal engagement in the evaluation. The program psychologist has a certification (PSYPACT) that is available to psychologists licensed in certain states that allows telehealth practice across state lines in locations that have adopted the legislation approving this (see https://www.psycpact.org). Patients seen via telehealth tend to be ones that have previously established care in the hospital system and determined to be low safety risk based on information in their medical record (i.e., no indication of previous suicidal ideation or other significant mental health problems).

More broadly, flexibility on the part of the psychologist and the team is also helpful in the rare cases where families express uncertainty or doubt about the need to see a psychologist, as for some families this may be perceived as creating an undesirable stigma for the patient. Prior to participating in the program, families are informed that psychology consultation is one of the components of the multidisciplinary evaluation. This is conveyed in a variety of ways including printed materials sent to the family in advance of their scheduled clinic date, description of clinic components on the program website, and verbally through families' conversations with the program coordinator and the nurse practitioner who manages the program. In the rare instances where families specifically inquire about the need to see psychology, typically the psychologist or nurse practitioner provides additional education about the role of psychology in this context, often via phone or face-to-face conversation on testing day. This conversation typically includes providing information about the high rate of psychological challenges seen in this population, related not only to mental health concerns but also to the significant burdens children and parents face in managing care for SV CHD. After sharing this information and listening to any concerns they may have, most families are agreeable.

In the current sample, results from the emotional/behavioral screening measures administered indicated that up to approximately one quarter of patients had elevated depression/anxiety symptoms and more than a third had elevated inattention symptoms. Furthermore, on the child self-report measure, over 20% of patients reported elevated levels of school problems, such as learning concerns and a generally negative attitude towards teachers and school. These findings are consistent with other research showing increased risk for neurocognitive and emotional/behavioral challenges compared to the general population and patients with other forms of CHD. Data from the quality-of-life measures completed by patients and their caregivers suggested mild to moderate impairments in this domain, consistent with findings from other samples (9).

As a secondary aim of the paper, exploratory analyses examined associations between scores on emotional/behavioral problems and quality of life scales. Patients with greater levels of inattention, depression/anxiety symptoms, and school problems had lower quality of life scores. These correlational analyses do not allow for determining causal links between these concerns, yet the associations suggest interrelatedness between psychological and health-related functioning for patients with FC. Although speculative, it is possible that children that reported lower health related quality of life may have poorer health overall requiring them to miss out on developmentally typical activities, including school and socialization with peers, which could in turn result in higher levels of depression or anxiety. It is also possible that the typical medical interventions used for staged palliation and the associated sequelae (e.g., early childhood stress during prolonged hospitalizations, lower brain oxygenation related to cardiopulmonary bypass in open heart surgery, lower resting oxygen saturations during sensitive periods of brain development) increase the risk for inattention, depression/anxiety symptoms, and school problems which then contribute to lower quality of life.

Future research could explore how specific physical health metrics and aspects of medical history might be related to certain psychological and quality of life issues commonly seen in these individuals. Prior studies have identified only a few medical factors that were associated with quality of life and emotional/behavioral functioning in individuals with FC. For example, a meta-analysis found that older age at the time of Fontan surgery was linked to poorer self-reported emotional functioning, and having a diagnosis of HLHS indicated greater likelihood of reporting problems with social functioning (3). Another study looked at psychological symptoms and results from cardiopulmonary exercise testing and found greater inattention symptoms were associated with lower peak VO2 as well as higher levels of anxiety were negatively correlated with heart rate recovery after exercise (10). Furthermore, poorer ventricular capacity was related to lower quality of life scores in a study of adolescents and adults with FC (11). However, other evidence suggests that even the most clinically well patients, those considered to be “Super Fontans” demonstrating normal performance on exercise testing metrics, do not report any meaningful differences in quality of life compared to those with poor exercise test performance (12). Taken together, these mixed and somewhat equivocal findings do not provide a clear understanding of how those with FC that have more psychological concerns differ from those who do not. Prospective research that includes thoughtful consideration about assessment methods for patient-reported outcomes are needed (13).

Although the primary goal of this paper is to describe our process for psychology consultation in the FORWARD Program, some limitations regarding the data reported are worth noting. The extent to which our findings are representative of the larger population of children and adolescents with FC in this age range is unclear, particularly as it relates to the homogeneity of racial/ethnic background of patients in our sample as well as the relatively lower rate of patients with Medicaid insurance compared to the general population of children in the United States, which is approximately 40% (14, 15). Furthermore, we do not report on background information of the caregivers who completed the screening measures in this sample, however, data on caregiver role (e.g., mother, father, etc.) is now being collected during administration. Lastly, while we aim to have data from the standardized psychological screening measures (i.e., BASC-3 and PCQLI) for all patients, results were only obtained for a portion of patients seen in clinic. Although reasons for this are unclear, this could be related to patient/family lack of understanding of the importance of completing them, challenges with health literacy, starting but not finishing them, or simply forgetting. We did have routine reminders in place for incomplete forms, including automated emails and verbal reminders from the psychologist during the consultation if they had not already been completed. As a result, any potential impact of the missing data on the findings reported in the current study cannot be ascertained.

Programs caring for patients with FC at different institutions may have a range of resources and support, or lack thereof, to implement emotional/behavioral health consultations as routine care. Although we suggest that a fully integrated care model is ideal, programs may wish to consider other models of care based on the resources available to them. For example, beginning with a pilot universal screening program to demonstrate the need and potential impact of comprehensive psychology services could allow advocacy for additional resources to support the unique behavioral health issues seen in this population. Another important resource is that our program is a part of the Fontan Outcomes Network (FON), a collaborative learning network and clinical registry with member clinicians, researchers, patients, and families dedicated to the goal of improving outcomes for individuals with FC through data sharing and research (https://www.fontanoutcomesnetwork.org) (16). FON has developed a standardized form for participating institutions that allows for more consistent data collection across sites, which assesses a variety of health domains and variables including emotional/behavioral health. After this data form was finalized, we made slight modifications to our program's approach to psychology consultation to be consistent with the standardized approach.

Future researchers and clinicians examining programs like the one described in this paper are encouraged to think innovatively and strategically about ways of demonstrating the efficacy or added benefit to patients and families as a result of receiving routine psychological consultation. Evidence is emerging that generally supports behavioral health integration into pediatric medical care as a means for improving quality and outcomes in other disease populations, such as better glycemic control in type 1 diabetes, reduced child emotional/behavioral problems, and higher scores on quality of life metrics for both patients and parents across populations (7). Given the high risk for psychological concerns in patients with FC compared to other pediatric chronic illnesses (2), it is possible for even greater benefit to emerge as a result of behavioral healthcare integration for this group.

## Data Availability

The datasets presented in this article are not readily available because due to legal and institutional policies around confidentiality of protected health information (PHI). However, portions of the data may be available in some circumstances upon reasonable request. Requests to access the datasets should be directed to Jack Rychik, MD, rychik@chop.edu.
